# Sterol Composition Modulates the Response of *Saccharomyces cerevisiae* to Iron Deficiency

**DOI:** 10.3390/jof7110901

**Published:** 2021-10-25

**Authors:** Tania Jordá, Nicolas Rozès, Sergi Puig

**Affiliations:** 1 Departamento de Biotecnología, Instituto de Agroquímica y Tecnología de Alimentos (IATA), Consejo Superior de Investigaciones Científicas (CSIC), 46980 Valencia, Spain; tajorsan@iata.csic.es; 2 Departament de Bioquímica i Biotecnología, Facultat d’Enologia, Universitat Rovira i Virgili, 43007 Tarragona, Spain; nicolasrozes@urv.cat

**Keywords:** baker’s yeast, *Saccharomyces cerevisiae*, iron deficiency, sterols, ergosterol, Upc2, Ecm22, Aft1

## Abstract

Iron is a vital micronutrient that functions as an essential cofactor in multiple biological processes, including oxygen transport, cellular respiration, and metabolic pathways, such as sterol biosynthesis. However, its low bioavailability at physiological pH frequently leads to nutritional iron deficiency. The yeast *Saccharomyces cerevisiae* is extensively used to study iron and lipid metabolisms, as well as in multiple biotechnological applications. Despite iron being indispensable for yeast ergosterol biosynthesis and growth, little is known about their interconnections. Here, we used lipid composition analyses to determine that changes in the pattern of sterols impair the response to iron deprivation of yeast cells. Yeast mutants defective in ergosterol biosynthesis display defects in the transcriptional activation of the iron-acquisition machinery and growth defects in iron-depleted conditions. The transcriptional activation function of the iron-sensing Aft1 factor is interrupted due to its mislocalization to the vacuole. These data uncover novel links between iron and sterol metabolisms that need to be considered when producing yeast-derived foods or when treating fungal infections with drugs that target the ergosterol biosynthesis pathway.

## 1. Introduction

Iron is an indispensable element for the large majority of living organisms because it functions as an essential cofactor in oxygen transport, respiration, and many metabolic processes, including lipid biosynthesis. Despite the iron abundance, the low solubility of its oxidized form (Fe^3+^) at physiological pH dramatically limits iron bioavailability. Thus, iron deficiency is a widely extended nutritional disorder affecting humans (predominantly children and women), animals, and crops [1,2]. Strategies to prevent and treat human iron deficiency include diet diversification, iron supplementation, and fortification of food with iron [3]. *Saccharomyces cerevisiae* is one of the most important microorganisms in biotechnology because it has been used since ancient times to obtain fermented foods (e.g., wine, beer, and bread) and, more recently, as a cell factory. Yeast itself is also consumed as a food supplement because it is especially rich in vitamins, proteins, and fiber. Importantly, iron-enriched yeasts can also be used to prevent and ameliorate iron deficiency symptoms in animals and humans [4,5,6].

Baker’s yeast is a reliable model organism to investigate the response of eukaryotes to iron limitation [7,8]. The response of *S. cerevisiae* to iron deficiency depends on the two partially overlapping Aft1 and Aft2 transcription factors. Upon iron deficit, these iron-sensing proteins accumulate in the nucleus, where they associate to iron-responsive promoter elements (FeRE) and promote the transcription of a set of genes known as the iron regulon, which includes the high-affinity iron acquisition complex *FET3*/*FTR1*, the iron-siderophore transporter *ARN2,* and the metalloreductase *FRE4*, whose main function consists in increasing the intracellular bioavailability of iron, and the mRNA-binding protein *CTH2*, which is implicated in remodeling iron metabolism [8].

**Figure 1:**
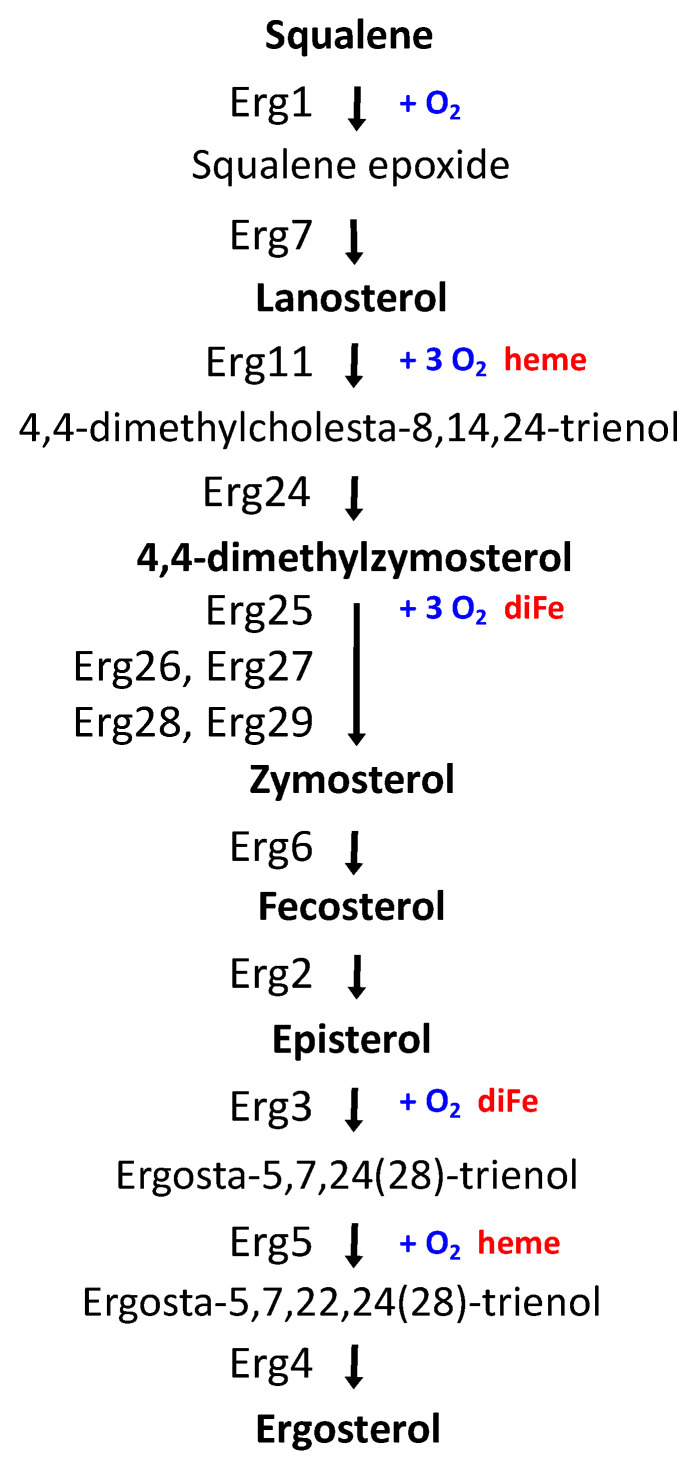
Late *S. cerevisiae* ergosterol biosynthetic pathway. Erg1: squalene monooxygenase. Erg7: lanosterol synthase. Erg11: lanosterol C-14 demethylase. Erg24: sterol C-14 reductase. Erg25: sterol C-4 methyloxydase. Erg26: sterol C-3 dehydrogenase. Erg27: sterol C-3 ketoreductase. Erg28 and Erg29: C-4 demethylation complex reaction. Erg6: sterol C-24 methyltransferase. Erg2: sterol C-8 isomerase. Erg3: sterol C-5 desaturase. Erg5: sterol C-22 desaturase. Erg4: sterol C-24 reductase. Sterol intermediates, enzymes, and their requirements of oxygen and iron are shown.

Iron is an indispensable cofactor in multiple yeast enzymes critical for lipid anabolic metabolism, such as (i) Δ9-fatty acid desaturase (Ole1) in the synthesis of unsaturated fatty acids (UFAs), (ii) lanosterol C-14 demethylase (Erg11), sterol C-4 methyloxydase (Erg25), sterol C-5 desaturase (Erg3), and sterol C-22 desaturase (Erg5), which are required for sterol production (Figure 1), and (iii) sphinganine C4-hydroxylase (Sur2), sphingolipid alpha-hydroxylase (Scs7), and 2-hydroxy fatty acid dioxygenase (Mpo1) in sphingolipid metabolism [9,10]. Thus, a decline in iron bioavailability causes a decrease in the levels of cellular UFAs, ergosterol, and sphingolipids, which affects yeast growth and nutritional quality [10,11]. The lipid composition of cellular membranes determines their physicochemical properties, which are critical to maintaining cell integrity and functionality. Ergosterol is the most abundant sterol compound in yeast cells, mostly present in the plasma membrane, the secretory pathway, and lipid particles [12]. In contrast, vacuoles are relatively poor in sterols but contain sterol intermediates absent in the plasma membrane, such as episterol, zymosterol, lanosterol, and fecosterol [12,13]. Dynamic changes in the sterol and fatty acid composition of biological membranes allow cells to adapt to metabolic remodeling and environmental stresses [14,15]. Consequently, the expression of multiple enzymes implicated in lipid biosynthesis is finely regulated. In particular, ergosterol biosynthesis (*ERG*) genes are controlled at the transcriptional level in response to changes in sterol levels and oxygen, which is an essential substrate for several enzymes in the late ergosterol synthesis pathway (Figure 1) [9]. In response to low oxygen, ergosterol synthesis decreases, and several *ERG* genes are activated by two partially redundant Zn_2_-Cys_6_ binuclear cluster transcription factors, denoted as Upc2 and Ecm22 [9]. Under normal growth conditions, the disruption of either *UPC2* or *ECM22* leads to minor changes in the profile of sterol species, whereas the double mutation of *UPC2* and *ECM22* results in decreased ergosterol levels [16]. Moreover, the deletion of any of the non-essential last five enzymes within the pathway, encoded by *ERG2* to *ERG6* genes, prevents the synthesis of ergosterol and causes the accumulation of sterol intermediate mixtures. In fungi, ergosterol has been recently shown to be indispensable for the maintenance of mitochondrial DNA [17]. Despite this, ergosterol synthesis is frequently suppressed or modified to optimize productivity, underestimating physiological consequences [15]. Importantly, multiple enzymatic steps within the late ergosterol biosynthesis pathway are used as targets to develop antifungal agents. For instance, azoles are widely used to inhibit lanosterol C-14 demethylase (Erg11), allylamines target squalene epoxidase (Erg1), morpholines preferentially inhibit sterol C-14 reductase (Erg24), and polyenes directly associate to cell surface ergosterol [9]. Thus, *S. cerevisiae* is used as a model system to study fungal susceptibility to azole drugs [18].

In this work, we used *upc2Δ/ecm22Δ* and *erg2Δ-erg5Δ* mutants to investigate how defects in ergosterol biosynthesis influence the adaptation of yeast to iron-limited conditions. Data support that yeast cells with defective sterol synthesis exhibit important growth defects in media with low iron bioavailability due to the impaired activation of the Aft1-dependent transcriptional response and altered sterol composition.

## 2. Materials and Methods

### 2.1. Yeast Strains, Plasmids, and Growth Conditions

The description of *S. cerevisiae* yeast strains and plasmids utilized in this work are shown in Table S1. To construct wild-type and *mga2Δ* strains with the genomic copy of *AFT1* tagged with the *GFP* epitope at the carboxyl terminus (SPY1212 and SPY1213 strains, respectively), we used an integrative PCR cassette generated with the pFA6a-GFP(S65T)-His3MX6 plasmid as a template and the oligonucleotides AFT1-F2 and AFT1-R1, as described in [19]. The correct integration was confirmed by PCR on genomic DNA using the oligonucleotides TermTEF:135F and Aft1+275-R (Table S2). Yeast was cultivated as previously described [20]. Iron deficiency (−Fe) was achieved by supplementation with 100 µM bathophenanthroline disulfonic acid disodium (BPS) (Sigma, Darmstadt, Germany), a Fe^2+^-specific chelator. In the specific case of wild-type (W303), *upc2Δ*, *ecm22Δ,* and *upc2Δecm22Δ*, cells were cultivated for 15 h to exponential phase in SC (+Fe) and then 100 µM BPS was added, and cells were cultivated for 9 additional hours (−Fe). Iron deficiency was achieved in 2% agar (Condalab, Madrid, Spain) solid SC (lacking specific requirements when necessary) by adding the Fe^2+^-specific chelator Ferrozine (Sigma). Spot assays were performed as described [20]. Ergosterol (ACROS Organics, Geel, Belgium) was added to 80 µg/mL final concentration and was solubilized in ethanol/Tergitol Nonidet P-40 (Sigma) (1:1, *v/v*).

### 2.2. RNA Analyses

Total RNA extraction and mRNA levels determination by RT-qPCR were performed as previously indicated [21]. Table S2 includes the primers used for RT-qPCR.

### 2.3. Determination of Total Fatty Acids and Sterols

Analysis of fatty acid methyl ester was performed as previously described [22]. For determining sterol cell composition, a pellet of 5·10^8^ cells, 5 mL of 12% (*w/v*) KOH in methanol was added to resuspend cells into glass tubes. Then, 1 mL pyrogallol (0.5%, *w/v*) in methanol and 10 μL α-cholestane (1 g/L, internal standard) were added. Samples were in a dry bath at 70 °C for 1 h. When samples were cooled, 1 mL of water was added, and then samples were extracted twice with 400 µL hexane. Tubs were centrifuged for 2 min at 3000× *g* between each extraction period. After that, the two organic phases were recuperated in microtubes, dried by nitrogen gas, resuspended with 100 μL hexane, and introduced into vial inserts. Gas chromatography mass spectrometry was carried out, as previously indicated [23]. After normalization with the α-cholestane area, chromatographic peak areas were used to calculate relative sterol abundance.

### 2.4. β-Galactosidase Assays

β-galactosidase activities were determined as previously indicated [24].

### 2.5. Fluorescence Microscopy

Cell quantification was performed as previously described [20].

### 2.6. Principal Component Analysis and Statistical Analyses

Principal component analyses (PCA) with an orthogonal Varimax rotation were performed to evaluate differences in sterol profiles between each strain under both iron sufficiency and deficiency conditions. Pearson correlation coefficient was used to establish the correlation between sterols, and the sampling adequacy was examined via the Kaiser–Meyer–Olkin (KMO) measurement. Statistical significance was assessed with tailed t-student tests. Significant differences among groups (*p*-value < 0.05) are indicated by different letters above bars.

## 3. Results

### 3.1. Full Activation of the Iron Regulon Requires Ergosterol Biosynthesis

**Figure 2:**
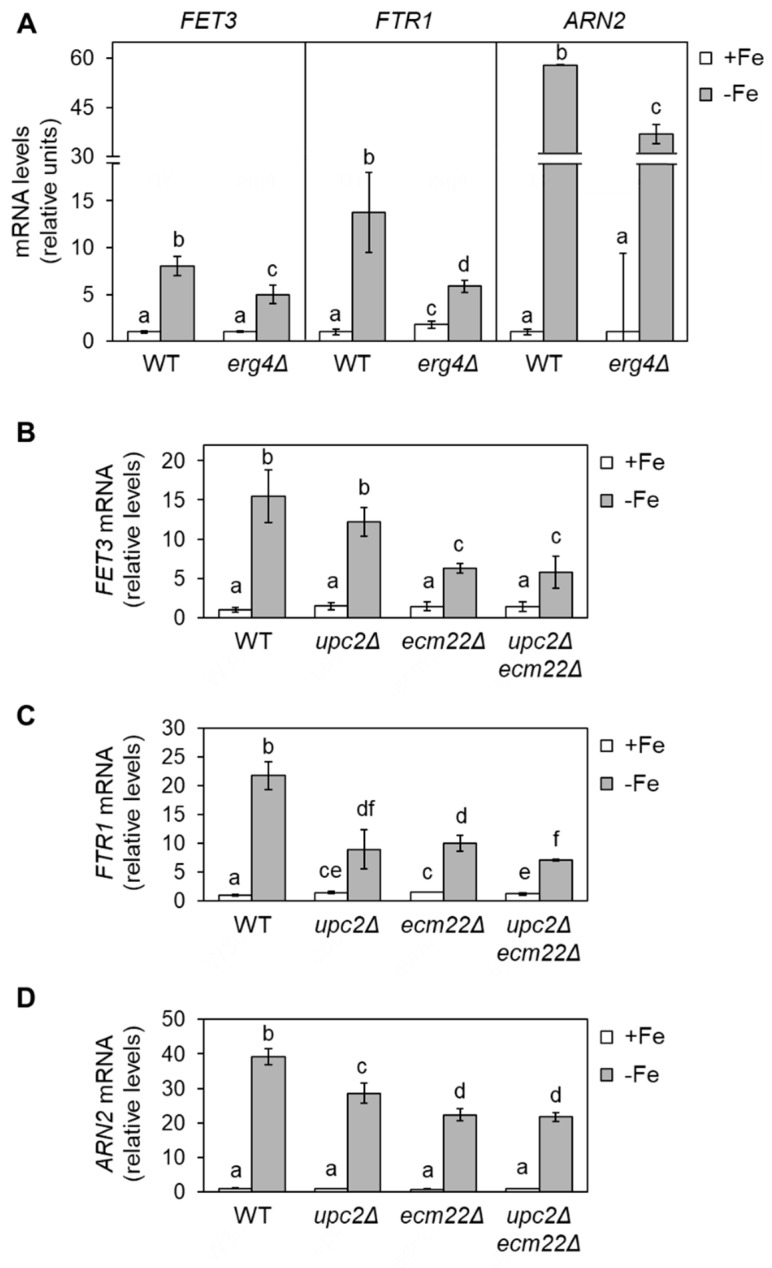
Defects in ergosterol biosynthesis limit the up-regulation of iron regulon genes during iron deficiency. (**A**) Wild-type (WT, BY4741) and *erg4Δ* cells were cultivated in SC at 30 °C in SC medium without (+Fe) or with 100 µM BPS (−Fe) for 6 h. (**B**–**D**) Wild-type (WT, W303), *upc2Δ*, *ecm22Δ,* and *upc2Δecm22Δ* cells were grown for 15 h to exponential phase in SC medium (+Fe) and then 100 µM BPS was added, and cells were cultivated for 9 h (−Fe). Total RNA was extracted, and mRNA levels of *FET3*, *FTR1,* and *ARN2* were determined by RT-qPCR as indicated in Material and Methods. Data were normalized to *PGK1* (**A**) or *ACT1* (**B**–**D**) mRNA levels. Data display the average and standard deviation (SD) of three biologically independent assays relative to wild-type cells in +Fe conditions. Different letters above bars indicate statistically significant differences (*p*-value < 0.05).

Given that sterols contribute to membrane fluidity and ergosterol biosynthesis is an iron-dependent metabolic pathway, we decided to address whether changes in ergosterol biosynthesis influence the response of yeast to iron depletion. Since the initial steps of the late ergosterol biosynthesis pathway are essential, we assayed single mutants in the last four enzymatic steps of the pathway including sterol C-8 isomerase (*ERG2*), sterol C-5 desaturase (*ERG3*), sterol C-22 desaturase (*ERG5*), and sterol C-24 reductase (*ERG4*) (Figure 1). We first checked the activation of several iron regulon genes implicated in iron uptake (*FET3*, *FTR1,* and *ARN2*) in an *erg4Δ* mutant, which lacks the final step in ergosterol synthesis. We grew wild-type and *erg4Δ* yeast cells under iron-sufficient (+Fe) and iron-deprived (−Fe) conditions and determined the expression levels of iron regulon genes by RT-qPCR. We observed that basal expression levels under iron-sufficient conditions were very low and indistinguishable between wild-type and *erg4Δ* cells (Figure 2A). Importantly, *erg4Δ* mutants displayed a defect in the activation of iron regulon genes upon iron depletion (Figure 2A). Similar to *erg4Δ* mutants, *erg2Δ*, *erg3Δ,* and, to a minor extent, *erg5Δ* mutants exhibited a diminished expression of multiple members of the iron regulon when iron bioavailability was restricted (Figure S1). These results suggest that ergosterol biosynthesis is important for the appropriate expression of the iron regulon in response to iron starvation.

To further assess whether ergosterol biosynthesis influences the activation of the components of the iron regulon, we tested mutants in Upc2 and Ecm22 transcription factors (*upc2Δ*, *ecm22Δ,* and *upc2Δecm22Δ*), which are required for the expression of *ERG* genes [9]. As previously reported, *FET3*, *FTR1*, *ARN2, CTH2,* and *FRE4* mRNA levels increased in iron-depleted wild-type cells (Figure 2 and Figure S1). The up-regulation was importantly affected in *upc2Δecm22Δ* double mutant cells but also diminished in both *upc2Δ* and *ecm22Δ* single mutants (Figure 2 and Figure S1). The lower iron regulon activation observed in *erg2Δ-erg5Δ* and *upc2Δ*/*ecm22Δ* mutant cells was not due to a decrease in UFA synthesis since there was no consistent variation in the levels of *OLE1* mRNA or UFAs/SFAs ratio with respect to wild-type cells (Figure S2). These results strongly suggest that the integrity and proper expression of the ergosterol biosynthetic pathway are critical for the activation of the yeast iron deficiency response.

**Figure 3:**
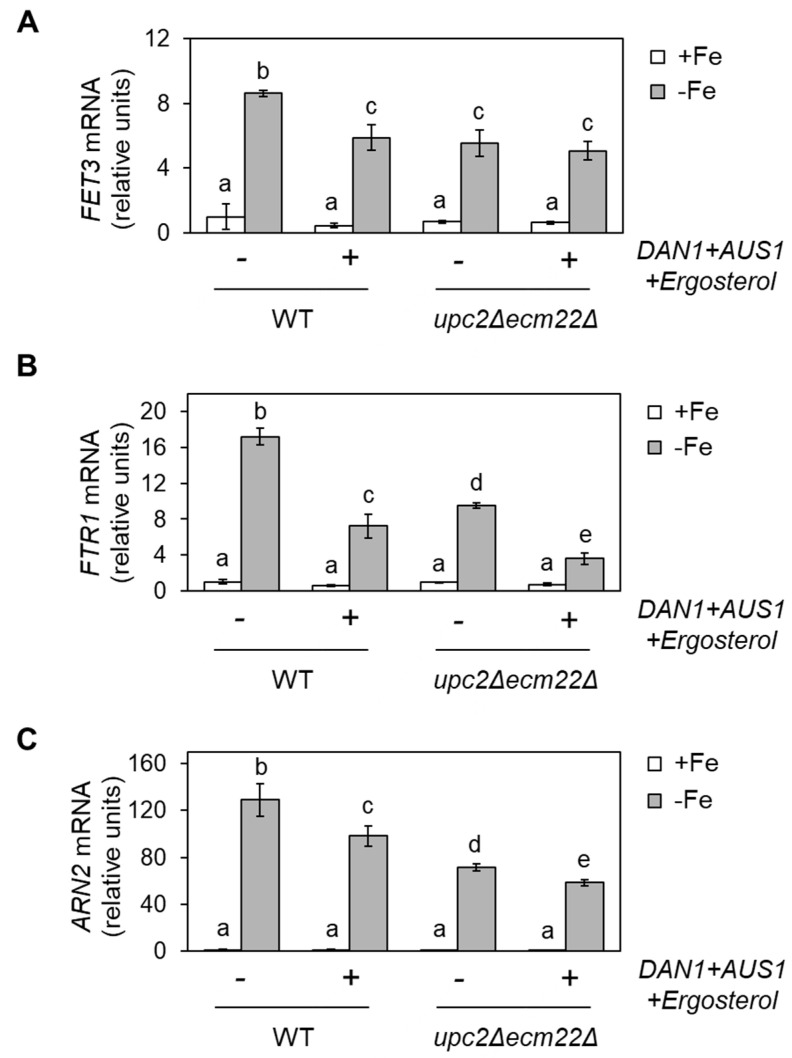
Supplementation with ergosterol decreases the iron regulon activation upon iron deficiency. Wild-type (WT, W303) and *upc2Δecm22Δ* cells were transformed with both pNEV-*DAN1* and YEp13-*AUS1* plasmids or with the two corresponding empty vectors, pNEV, and YEp13. Then, transformed cells were cultivated at 30 °C for 6 h in SC-Ura-Leu medium without (+Fe) or with 100 µM BPS (−Fe). Then, 80 μg/mL ergosterol was added to cells expressing *DAN1* and *AUS1.* Total RNA was extracted, and *FET3* (**A**), *FTR1* (**B**), and *ARN2* (**C**) mRNA levels normalized with *ACT1* mRNA were determined by RT-qPCR. Data show the average and SD of three independent experiments relative to wild-type cells grown in +Fe conditions. Significant differences are indicated above bars (*p*-value < 0.05).

We hypothesized that ergosterol supplementation could rescue the iron regulon activation defect displayed by mutants in *ERG* genes expression. It has been previously described that yeast cells only acquire extracellular sterols in anaerobiosis [9]. However, the overexpression of *AUS1* and *DAN1* genes slightly increases sterol acquisition under aerobic conditions [25]. Thus, as previously reported, we co-transformed wild-type and *upc2Δecm22Δ* cells with two plasmids, each containing either *DAN1* or *AUS1* under the control of the *PMA1* constitutive promoter or the two corresponding empty vectors [25]. Then, we determined the expression of members of the iron regulon under +Fe and −Fe conditions in the absence or after the addition of ergosterol to the growth medium. We first observed that the activation of the iron regulon by iron depletion in wild-type cells overexpressing *AUS1*/*DAN1* and supplemented with ergosterol was not as strong as in the original wild-type strain (Figure 3). Regarding *upc2Δecm22Δ* cells, we observed that ergosterol supplementation did not rescue the defective iron activation of *FET3*, *ARN2,* and *FTR1* genes (Figure 3).

### 3.2. Iron Deficiency Modifies the Sterol Profile of Deletion Mutants in UPC2 and ECM22 Genes

**Figure 4:**
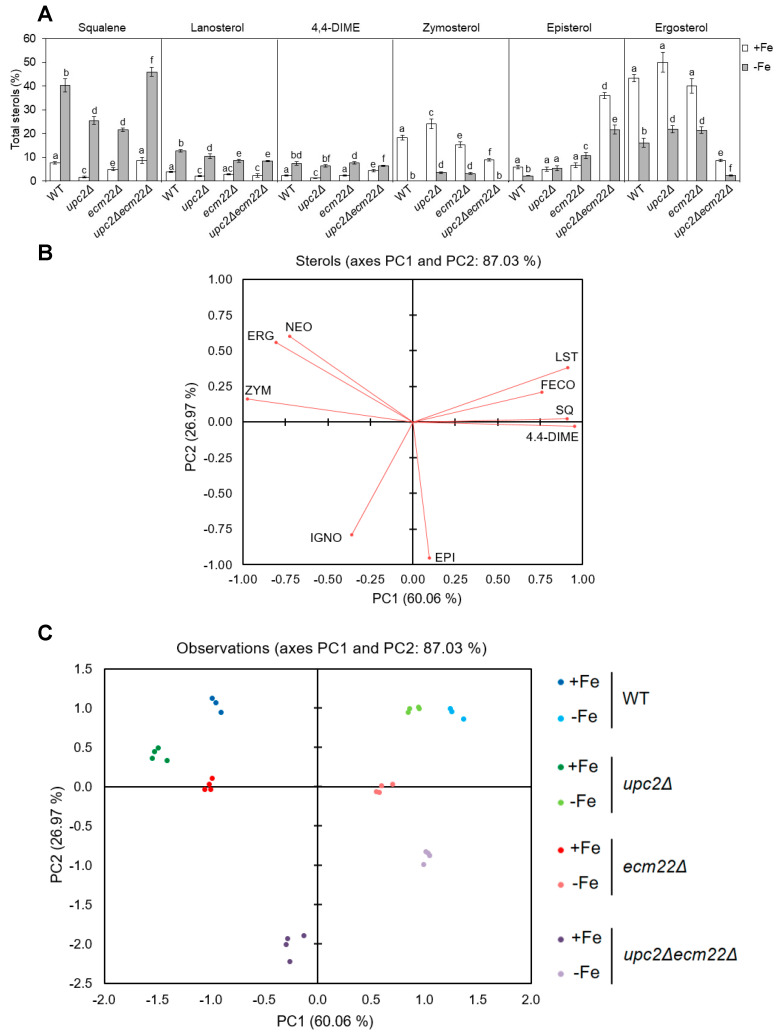
Iron deficiency modifies the sterols profile of *upc2* and *ecm22* mutant cells. Yeast wild-type (WT, W303), *upc2Δ*, *ecm22Δ,* and *upc2Δecm22Δ* cells were cultivated at 30 °C for 8 h in SC with 20 μM FAS (+Fe) or 100 μM BPS (−Fe). Then, sterol intermediates were extracted and quantified as indicated in the Material and Methods section. (**A**) Levels of intermediate sterols in each strain under both +Fe and −Fe conditions, represented as a percentage of the total sterol content. Squalene, lanosterol, 4,4-dimethylzymosterol (4,4-DIME), zymosterol, episterol, and ergosterol relative levels are represented. Data show the average and SD of four independent experiments. Different letters represent statistically significant differences between each strain for a specific sterol (*p*-value < 0.05). (**B**,**C**) Principal component analysis (PCA). Biplots of varimax rotated PCA for each strain on which sterols variables (**A**) and observations (**B**) are plotted. SQ, squalene; LST, lanosterol; 4,4-DIME, 4,4-dimethylzymosterol; ZYM, zymosterol, FECO, fecosterol, EPI, episterol, ERG, ergosterol; IGNO, ignosterol; NEO, neoergosterol.

Since ergosterol biosynthesis depends on iron and Upc2/Ecm22 at different enzymatic steps, we decided to characterize the sterol pattern of wild-type, *upc2Δ*, *ecm22Δ,* and *upc2Δecm22Δ* cells cultivated in +Fe and −Fe conditions. First, we represented the relative abundance of sterol species in all strains (Figure 4A and Figure S3). Consistent with the iron dependence of Erg11 and Erg25 enzymes, all iron-depleted cells exhibited an accumulation of early intermediates (squalene, lanosterol, and 4,4-dimethylzymosterol) and a reduction in late sterol compounds (zymosterol, episterol, and ergosterol) compared with iron replete cells (Figure 4 and Figure S3). To further dissect complex sterol patterns, data were also analyzed by principal component analysis (PCA). Nine quantified sterols were used to identify the two principal components, which accounted for 87.03% of the total variance (60.06% and 26.97% for PC1 and PC2, respectively). Squalene, lanosterol, 4,4-dimethylzymosterol, zymosterol, ergosterol, and neoergosterol species mainly contributed to the first component, while episterol and ignosterol had a higher effect on the second component (Figure 4B). The Kaiser–Meyer–Olking measure of sampling adequacy was 0.712, which indicates that the degree of joint relationship between the sterol variables is adequate. According to PCA, iron-depleted cells were clearly different from iron-sufficient cells, and *upc2Δecm22Δ* mutant was markedly more divergent in both iron status (Figure 4C). The content of zymosterol, ergosterol, and neoergosterol were effective for the discrimination of iron-sufficient wild-type, *upc2Δ,* and *ecm22Δ* cells (Figure 4B,C). Nevertheless, *upc2Δ* and *ecm22Δ* cells showed lower levels of squalene and slightly higher levels of last sterol species, including episterol and ergosterol, than wild-type cells upon iron depletion (Figure 4A). On the other hand, iron-depleted wild-type and simple mutant cells were characterized by their higher levels of squalene, lanosterol, 4,4-dimethylzymosterol, and fecosterol, which are more similar between wild-type and *upc2Δ* cells than *ecm22Δ* cells (Figure 4B,C). The *upc2Δecm22Δ* mutant was distinguished by high levels of episterol and low ergosterol abundance, as compared to other cells (Figure 4A). Other minor changes in sterol profiles were also detected (Figure S3). Although strain-specific features were observed, this overall analysis indicates that *upc2Δecm22Δ* double mutant possesses a sterol distribution pattern that significantly differs from wild-type, *upc2Δ,* and *ecm22Δ* single mutants.

### 3.3. Growth in Iron-Deficient Conditions Requires Ergosterol Biosynthesis

**Figure 5:**
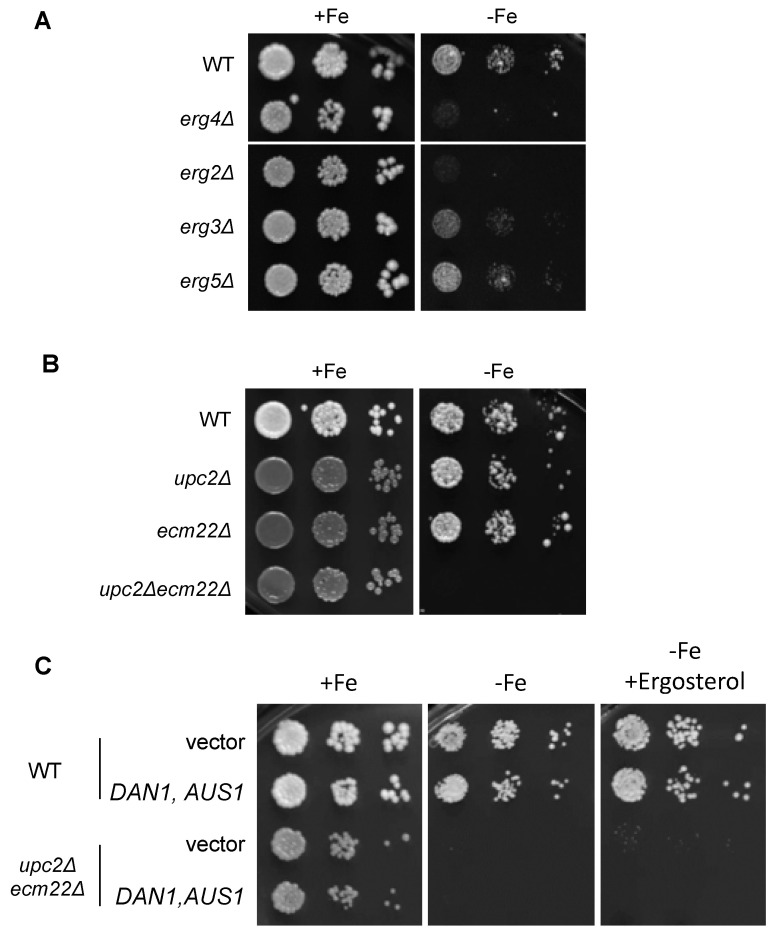
Ergosterol biosynthesis is essential for a proper adaptation of yeast to iron deficiency. (**A**) Single mutants of non-essential *ERG* genes exhibit growth defects under iron depletion. Wild-type (WT, BY4741), *erg4Δ*, *erg2Δ*, *erg3Δ,* and *erg5Δ* cells were spotted in 10-fold serial dilutions on SC (+Fe) and SC supplemented with 600 μM Ferrozine (−Fe). (**B**) The simultaneous deletion of *UPC2* and *ECM22* genes limits growth under iron starvation. Wild-type (WT, W303), *upc2Δ*, *ecm22Δ,* and *upc2Δecm22Δ* cells were assayed for growth in SC without (+Fe) and with 400 μM Ferrozine (−Fe). (**C**) Ergosterol supplementation does not rescue the growth defect of *upc2Δecm22Δ* mutants in iron-deficient media. Wild-type (WT, W303) and *upc2Δecm22Δ* cells cotransformed with both pNEV-DAN1 and YEp13-AUS1 plasmids or with the empty vectors pNEV and YEp13 were assayed for growth in SC-Ura-Leu (+Fe), SC-Ura-Leu with 300 μM Ferrozine (−Fe), and SC-Ura-Leu with 300 μM Ferrozine and 80 μg/mL ergosterol (−Fe+Ergosterol). In all cases, plates were incubated for 3–4 days at 30 °C and then photographed. A representative experiment of two different assays is shown.

To assess the implication of sterols in yeast adaptation to iron deficiency, we investigated growth in low-iron conditions of viable *ERG* gene deletion mutants, which show defects in ergosterol synthesis and accumulate sterol intermediates [15]. A significant growth defect in iron-deficient conditions was observed in strains deleted for *ERG2*, *ERG3*, *ERG4*, and, to a lesser extent, *ERG5* (Figure 5A). To further ascertain whether proper ergosterol biosynthesis was important under low-iron conditions, we assayed growth of wild-type, *upc2Δ*, *ecm22Δ*, and *upc2Δecm22Δ* cells under iron deprivation. All strains were able to grow in iron replete conditions (Figure 5B), whereas the *upc2Δecm22Δ* double mutant was completely unable to survive in an iron-deficient medium (Figure 5B). In contrast, single *upc2Δ* and *ecm22Δ* cells did not show growth defects under iron deprivation compared to the wild-type strain (Figure 5B), suggesting that defects in iron regulon activation are not sufficient to limit growth. Then, we attempted to rescue the phenotypic defects displayed by *upc2Δecm22Δ* mutant in iron-limited conditions by ergosterol supplementation (in combination with *DAN1* and *AUS1* overexpression). However, ergosterol was not able to rescue *upc2Δecm22Δ* growth in iron-depleted conditions (Figure 5C). Altogether, these results suggest that the combination of defects in the activation of the iron regulon and alterations in the levels of ergosterol and/or specific biosynthetic intermediates lead to growth defects under low iron bioavailability conditions.

### 3.4. Upc2 and Ecm22 Facilitate Transcription upon Iron Deficiency through Iron Regulatory Elements

**Figure 6:**
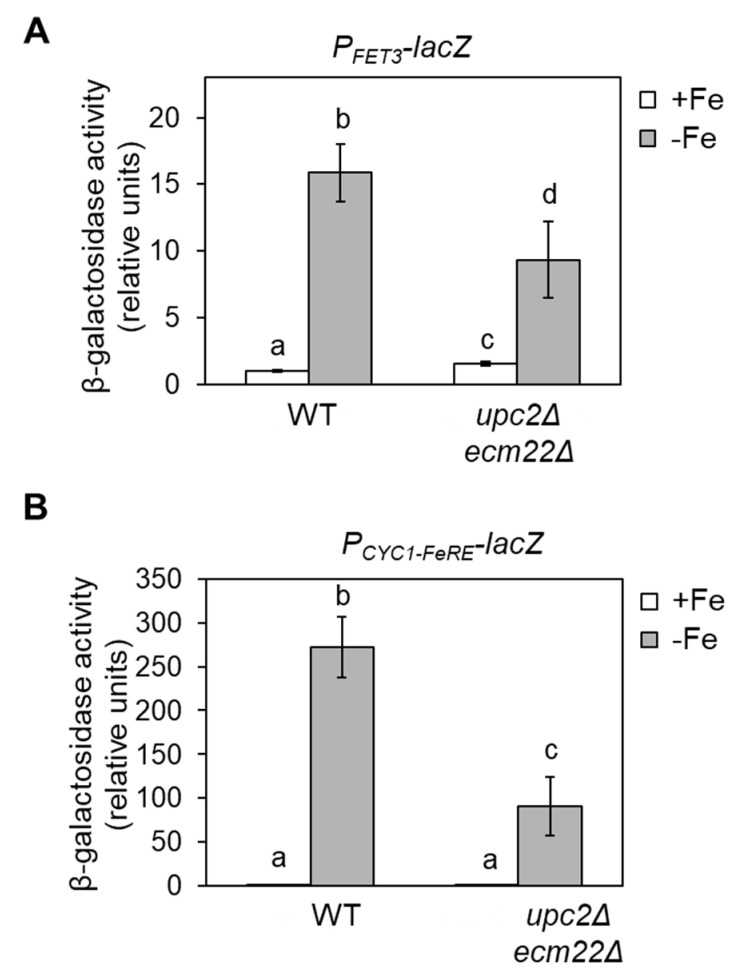
Upc2/Ecm22 regulation via promoter iron-regulatory elements. (**A**) Upc2/Ecm22-dependent activation of *FET3* upon iron deficiency occurs through its promoter. Wild-type (WT, W303) and *upc2Δecm22Δ* cells expressing *P_FET3_-lacZ* were grown at 30 °C in SC-Ura medium without (+Fe) or with 100 μM BPS (−Fe) for 6 h, and β-galactosidase activity was determined. Significant differences are represented by different letters above bars (*p*-value < 0.05). (**B**) FeRE sites are required for Upc2/Ecm22-mediated induction of transcription. Wild-type (WT, W303) and *upc2Δecm22Δ* cells expressing *P_CYC1-FeRE_–lacZ* were grown and analyzed as indicated in panel A. Data show the average and SD of three biologically independent experiments as compared to wild-type (+Fe) samples. Letters above bars indicate significant differences (*p*-value < 0.05).

To address whether the defect in the iron deficiency response observed in *upc2Δecm22Δ* cells occurred at the transcriptional level, we performed β-galactosidase activity assays with wild-type and *upc2Δecm22Δ* cells transformed with a plasmid containing *FET3* promoter fused to the *lacZ* coding sequence (*P_FET3_-lacZ*) and grown under both +Fe and −Fe conditions. Analogously to native *FET3* transcript levels, the expression of *P_FET3_-lacZ* was induced in iron-limited wild-type cells, whereas its induction significantly diminished in *upc2Δecm22Δ* cells (Figure 6A). To investigate the involvement of iron regulatory elements (FeREs) to which Aft1 transcription factor associates, we used a plasmid that expressed *lacZ* under the control of a basal *CYC1* promoter fused to two FeRE sequences (*P_CYC1-FeRE_-lacZ*). As previously reported [26], CYC1-fused FeRE sites trigger transcriptional activation in response to iron in wild-type cells (Figure 6B). However, up-regulation of *lacZ* via FeRE did not occur in *upc2Δecm22Δ* cells (Figure 6B). These results strongly suggest that Upc2 and Ecm22 modulate the activation of the iron regulon upon iron depletion via Aft1-binding sites.

### 3.5. Upc2 and Ecm22 Are Required for Appropriate Aft1 Protein Localization

**Figure 7:**
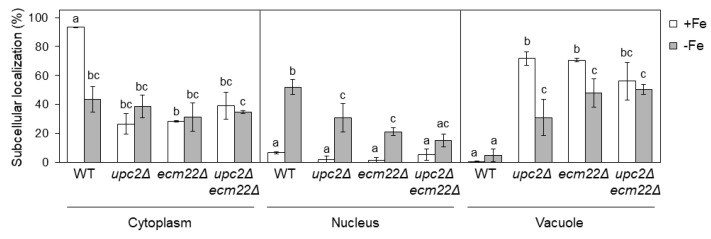
Upc2 and Ecm22 are necessary for the correct localization of Aft1 protein. Quantitative analysis of Aft1 subcellular localization patterns in wild-type (WT, W303), *upc2Δ*, *ecm22Δ*, and *upc2Δecm22Δ* cells containing JK1346 (pRS426-GFP-AFT1) plasmid. Cells were cultivated as indicated in Figure 6 and visualized under Nomarski (DIC) and GFP fluorescence optics. More than 100 cells were counted for at least three different experiments. GFP-Aft1 distribution patterns were considered as cytoplasmic, nuclear or vacuolar. Average and SD were represented. Statistical analysis has been performed independently for cytoplasm, nucleus, and vacuole, and different letters above bars of each panel indicate significant differences (*p*-value < 0.01).

In iron-sufficient conditions, the Aft1 transcription factor shuttles between the nucleus and the cytosol. However, in response to low iron, Aft1 protein accumulates in the nucleus and activates the expression of iron regulon genes [27,28]. We have recently reported that Aft1 protein predominantly mislocalizes to the vacuole in iron-depleted cells with defects in UFAs synthesis, achieved by deletion of the transcriptional factor Mga2 [20]. To ascertain whether Aft1 localization was altered by changes in sterol composition, we studied the subcellular localization of a functional GFP-Aft1 fusion protein in *upc2Δ*, *ecm22Δ,* and *upc2Δecm22Δ* mutants under +Fe and −Fe conditions. Under iron-sufficient conditions, we observed that most Aft1 protein in wild-type cells was cytoplasmic, whereas it accumulated in the vacuole of *upc2Δ, ecm22Δ,* and *upc2Δecm22Δ* cells (Figure 7). Opposite to *upc2Δ* and *ecm22Δ* mutants, we did not previously perceive significant differences in Aft1 subcellular localization between wild-type and *mga2Δ* mutants under iron-sufficient [20]. Since our previous Aft1 localization experiments with *mga2Δ* cells were performed expressing GFP-Aft1 in a high-copy plasmid expression system, we decided to check localization by epitope tagging genomic Aft1 with GFP at its carboxyl terminus. As described for sterol-defective mutants, genomically expressed Aft1-GFP was notably localized to the vacuole in *mga2Δ* under iron-sufficient conditions as compared to wild-type cells (Figure S4). These results indicate that defects in either UFA or ergosterol synthesis lead to vacuolar accumulation of the Aft1 transcription factor in iron-sufficient conditions.

As expected, nuclear Aft1 localization increased when wild-type cells were grown in low-iron conditions (Figure 7). Conversely, *upc2Δ, ecm22Δ,* and *upc2Δecm22Δ* mutants presented a defective Aft1 nuclear accumulation upon iron deficiency mostly due to its vacuolar location (Figure 7). Particularly, iron deficiency slightly decreased Aft1 vacuolar localization of *upc2Δ* and *ecm22Δ* cells and increased its nuclear location, but it did not alter Aft1 localization in the double *upc2Δecm22Δ* mutant (Figure 7). Altogether, these results indicate that Aft1 protein constitutive shuttling and its nuclear accumulation upon iron scarcity depends on proper UFA and sterol cellular content; otherwise, Aft1 accumulates into the vacuole leading to a defect in iron regulon activation. We also observed that *upc2Δ, ecm22Δ,* and *upc2Δecm22Δ* mutants displayed slightly lower *AFT1* mRNA levels than wild-type cells under iron-deficient conditions (Figure S1D), which could contribute to their Aft1-associated phenotypes.

### 3.6. upc2Δecm22Δ Phenotypes Are Rescued by a Constitutively Nuclear and Active Aft1 Protein

**Figure 8:**
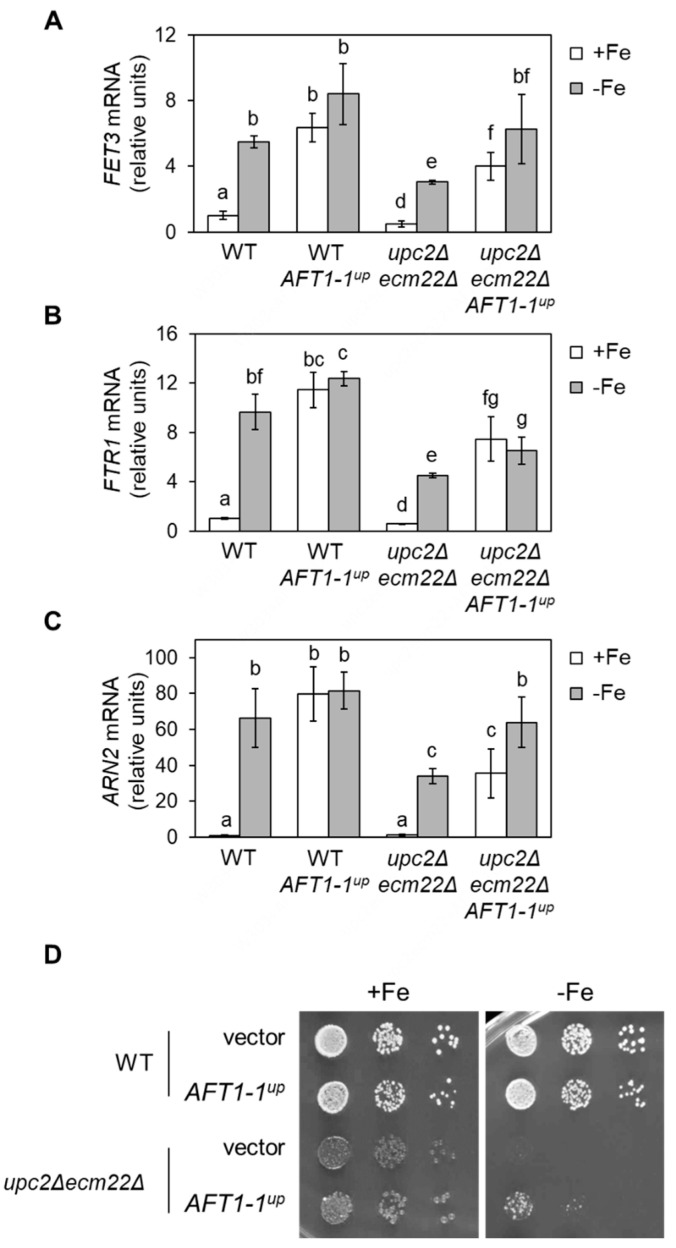
The phenotypes of *upc2Δecm22Δ* mutants under iron deficiency are partially rescued by the *AFT1-1^up^* allele. Wild-type (WT, W303) cells and the *upc2Δecm22Δ* mutant were transformed with either pRS416 (vector) or pRS416-AFT1-1^up^-12HA (*AFT1-1^up^*) plasmids. (**A**–**C**) *AFT1-1^up^* allele rescues the activation of the iron regulon. Cells were grown as in Figure 6. RNA was extracted, and mRNA levels of *FET3*, *FTR1,* and *ARN2* normalized to *ACT1* were determined by RT-qPCR. Data indicate the average and SD of three biologically independent experiments (*p*-values < 0.05). (**D**) The growth defect of *upc2Δecm22Δ* mutants under iron scarcity is partially rescued by the *AFT1-1^up^* allele. Growth in SC-Ura (+Fe) and SC-Ura with 300 μM Ferrozine (−Fe) was tested. A representative photograph of three biologically independent experiments is displayed.

The constitutively active *AFT1-1^up^* allele [29] was able to bypass the problems displayed by mutants with defects in UFA synthesis under iron starvation [20]. Thus, we attempted to rescue the misregulation of iron regulon genes in *upc2Δecm22Δ* cells using an *AFT1-1^up^*-expressing plasmid. Wild-type and *upc2Δecm22Δ* cells expressing *AFT1-1^up^* were grown in +Fe and −Fe conditions, and the transcript level of iron regulon genes was determined and compared to the corresponding cells expressing only wild-type *AFT1*. In wild-type cells, *FET3*, *FTR1,* and *ARN2* transcript levels increased in response to iron limitation, but activation was defective in *upc2Δecm22Δ* mutant (Figure 8A–C). As expected, *AFT1-1^up^* raised iron regulon expression under iron sufficiency in both wild-type and *upc2Δecm22Δ* cells (Figure 8A–C). More importantly, the expression of the Aft1-1^up^ protein rescued the defect in iron regulon activation displayed by *upc2Δecm22Δ* mutants (Figure 8A–C). Then, we assayed whether *AFT1-1^up^* could also recover growth under iron-deficient conditions. Indeed, *AFT1-1^up^* slightly rescued *upc2Δecm22Δ* growth defect in iron-limited conditions (Figure 8D). These results indicate that Aft1 mislocalization and its consequent iron regulon activation defect contributes to limiting *upc2Δecm22Δ* adaptation to iron depletion.

## 4. Discussion

The yeast *S. cerevisiae* is widely used in food biotechnology. As for many other organisms, yeast growth is highly limited by iron bioavailability, a micronutrient that is necessary for many metabolic pathways, including the biosynthesis of the predominant yeast sterol, ergosterol (Figure 1). However, little is known about how changes in sterol profiles influence iron homeostasis. By using yeast mutants defective in ergosterol synthesis (*upc2Δ*/*ecm22Δ* and *erg2Δ*-*erg5Δ*), we have shown here that a functional ergosterol biosynthetic pathway is necessary for the appropriate activation of the Aft1-dependent iron-deficiency transcriptional response (Figure 2 and Figure S1). Previous data have shown that yeast *mga2Δ* mutants display defects in the activation of iron regulon genes and an impairment in UFA biosynthesis [20]. However, we could discard an indirect effect of impaired ergosterol biosynthesis on UFA metabolism or *OLE1* expression (Figure S2). Remarkably, whereas supplementation with UFAs (linoleic acid) was sufficient to restore the iron deficiency response of *mga2Δ* cells [20], no effect was observed when ergosterol was added to *upc2Δecm22Δ* cells (Figure 3). Sterol composition revealed that the defect in iron regulon activation was not only due to decreased levels of ergosterol because *upc2Δ* and *ecm22Δ* single mutants displayed gene expression defects despite not having lower ergosterol levels than wild-type cells (Figure 2 and Figure 5). These results suggest that an adequate balance of sterol species is required for full activation of the iron regulon when the iron is scarce. Ergosterol is typically used as a supplement to the medium in biotechnology processes in which optimal oxygenation is not possible, such as alcoholic fermentation, since ergosterol biosynthesis requires oxygen at multiple enzymatic steps (Figure 1). Here, we observed that ergosterol supplementation also limits the transcriptional activation of iron regulon genes in wild-type cells (Figure 3), which could limit the response to iron deficiency. Therefore, special care should be taken on iron bioavailability to ensure yeast cell growth.

Surprisingly, not all yeast ergosterol mutants with impaired activation of the iron regulon exhibited growth defects in iron-deficient conditions. Whereas *upc2Δ* and *ecm22Δ* single mutants grew perfectly in solid media with limited availability of iron, *upc2Δecm22Δ* double mutant did not grow at all. *upc2Δecm22Δ* mutants contain significantly lower levels of ergosterol than *upc2Δ* and *ecm22Δ* cells, but growth rescue with ergosterol supplementation was unsuccessful (Figure 5C). It is possible that not sufficient ergosterol is entering *upc2Δecm22Δ* cells. An alternative explanation could be that, opposite to *upc2Δ* and *ecm22Δ* cells, *upc2Δecm22Δ* double mutant cells seem to possess a strong defect in the expression of *ERG* genes within the last enzymatic steps (*ERG2*-*ERG5*) since they accumulate high amounts of the initial substrate of these enzymes, episterol, in addition to alterations in other sterol intermediates (Figure 4 and Figure S3). In this sense, *upc2Δecm22Δ* mutant could behave as *erg2Δ*-*erg5Δ* mutants, which exhibit growth defects in iron-deficient conditions (Figure 5A). Due to the fact that Erg2-Erg5 enzymes recognize a broad range of sterols as substrates, their corresponding *erg* mutants, and potentially *upc2Δecm22Δ* cells, accumulate particular sterol mixtures instead of only increasing the concentration of their substrate in the pathway [15], leading to growth defects. Therefore, an altered balance of sterol species, the accumulation of toxic intermediate species or decreased ergosterol levels could be responsible for the growth defect of *upc2Δecm22Δ* cells in iron-deficient conditions.

Previous studies have also reported links between sterol and iron metabolisms. For instance, deletion of the *S. cerevisiae* heme-binding protein Dap1, which participates in the function of the lanosterol demethylase Erg11, leads to growth defects under iron-limiting conditions [30]. Yeast *dap1∆* mutant accumulates squalene, lanosterol, and episterol [31] and exhibits a reduction in iron regulon activation [30,31]. It is possible that Aft1 also localizes to vacuoles in *dap1∆* mutants. Interestingly, *ERG11* overexpression suppresses *dap1∆* low-iron sensitivity despite the small effect on ergosterol levels [31,32]. Specific yeast *erg25* mutants cause growth defects in low-iron conditions, and *erg29* mutants display defects in the assembly of iron–sulfur clusters and accumulation of iron in mitochondria [33,34]. By contrast, the overexpression of *ERG* genes also slowed or completely inhibited the growth in iron-limited media, probably due to sterol imbalances [35]. Similar results have been reported in other fungi. The disruption of *UPC2* in the fungal commensal-pathogen *Candida albicans*, which does not have an *ECM22* paralog, has been shown to preclude activation of iron-related genes and limit growth in low-iron conditions [36]. In addition, *Candida glabrata* iron acquisition genes are down-regulated by chemical inhibition of Erg11 with fluconazole [37].

In addition, to modulate the fluidity of lipid membranes, sterols also participate in other critical processes including endocytosis and vesicle formation, protein sorting, organization of the cytoskeleton, and mating. Thus, viable *erg* mutants exhibit defects in particular vesicular transport pathways and vacuolar fragmentation [15]. As a consequence, when yeast strains with altered sterol content are used as microbial cell factories to produce heterologous membrane-associated proteins, special attention should be taken to verify the proper localization of the expressed protein. Subcellular localization studies performed here with *upc2Δecm22Δ* cells have unveiled that the Aft1 transcription factor mislocalizes to the vacuole, preventing nuclear transcriptional activation upon iron depletion (Figure 7). Remarkably, the Aft1 protein mislocalization occurs under both iron-sufficient and iron-limited conditions in cells with either altered ergosterol or UFA biosynthesis (Figure 7 and Figure S5). The addition of ergosterol biosynthesis inhibitors, such as fluconazole or terbinafine, to *S. cerevisiae* cultures has been shown to limit the nuclear accumulation of Aft1 protein [38]. Despite the molecular bases for this mislocalization having not been deciphered, these data indicate that lipid composition is critical for the proper trafficking of membrane-associated proteins but also for the adequate translocation of soluble proteins, such as the Aft1 transcription factor, to the nucleus. Importantly, *upc2Δecm22Δ* growth defect in iron deficiency is partially rescued by the constitutively active *AFT1-1^UP^* allele (Figure 8), which indicates that impaired localization of Aft1 and defective iron regulon expression contribute, in addition to the unbalance of sterols, to growth problems in iron-deficient media. We cannot discard a direct regulation of Upc2 or Ecm22 transcription factors on particular members of the iron regulon. For instance, *ARN2* and *CTH2* genes contain potential sterol regulatory elements within their promoter regions that could facilitate Upc2/Ecm22 binding. Altogether, these data indicate that changes in UFA and sterol biosynthesis can lead to defects in the response of yeast cells to iron deficiency and cause important growth defects.

## 5. Conclusions

The deletion of non-essential genes (*ERG2*-*ERG5*) within the late ergosterol pathway is a common strategy to optimize the production of chemicals in *S. cerevisiae* cells. However, these mutants display altered plasma membrane lateral compartmentation dynamics, defective trafficking and vacuolar fusion, and reduced tolerance to micotoxic compounds and to multiple stresses, including high ethanol, oxidative and osmotic stress [15,39]. In this study, we report that defects in sterol biosynthesis also led to an impaired response to iron deficiency, which is an extremely frequent condition that should be considered when using this yeast in food biotechnology or as a microbial cell factory. Moreover, ergosterol biosynthesis is an iron-dependent pathway that is frequently used as a target for antifungal treatments. Thus, special care should be taken to evaluate fungal iron homeostasis when ergosterol-targeted drugs are used for the treatment of fungal infections.

## Data Availability

The data presented in this study are openly available in Digital CSIC (https://digital.csic.es) at DOI: http://dx.doi.org/10.20350/digitalCSIC/14003.

## Supplementary Materials

The following are available online at https://www.mdpi.com/article/10.3390/jof7110901/s1. Figure S1. Mutants in non-essential ergosterol biosynthesis genes display defects in iron regulon activation upon iron deficiency. Figure S2. The limitation of iron regulon activation in cells with defects in ergosterol biosynthesis is not due to a decrease in UFA synthesis. Figure S3. Iron deficiency modifies the sterols profile of Upc2/Ecm22 mutants. Figure S4. The localization of Aft1 protein is altered in *mga2Δ* mutants grown in iron-sufficient conditions. Table S1. Description and source of yeast strains and plasmids used in this work. Table S2. Oligonucleotides used for RT-qPCR in this work.
