# Supplementary material for: Sterol Composition Modulates the Response of Saccharomyces cerevisiae to Iron Deficiency
Source: J Fungi (Basel). 2021 Oct 25;7(11):901. doi: 10.3390/jof7110901 (PMC8620032; doi:10.3390/jof7110901)
Supplement: Supplementary file 1 [file jof-07-00901-s001.zip › jof-1405816-supplementary.pdf]

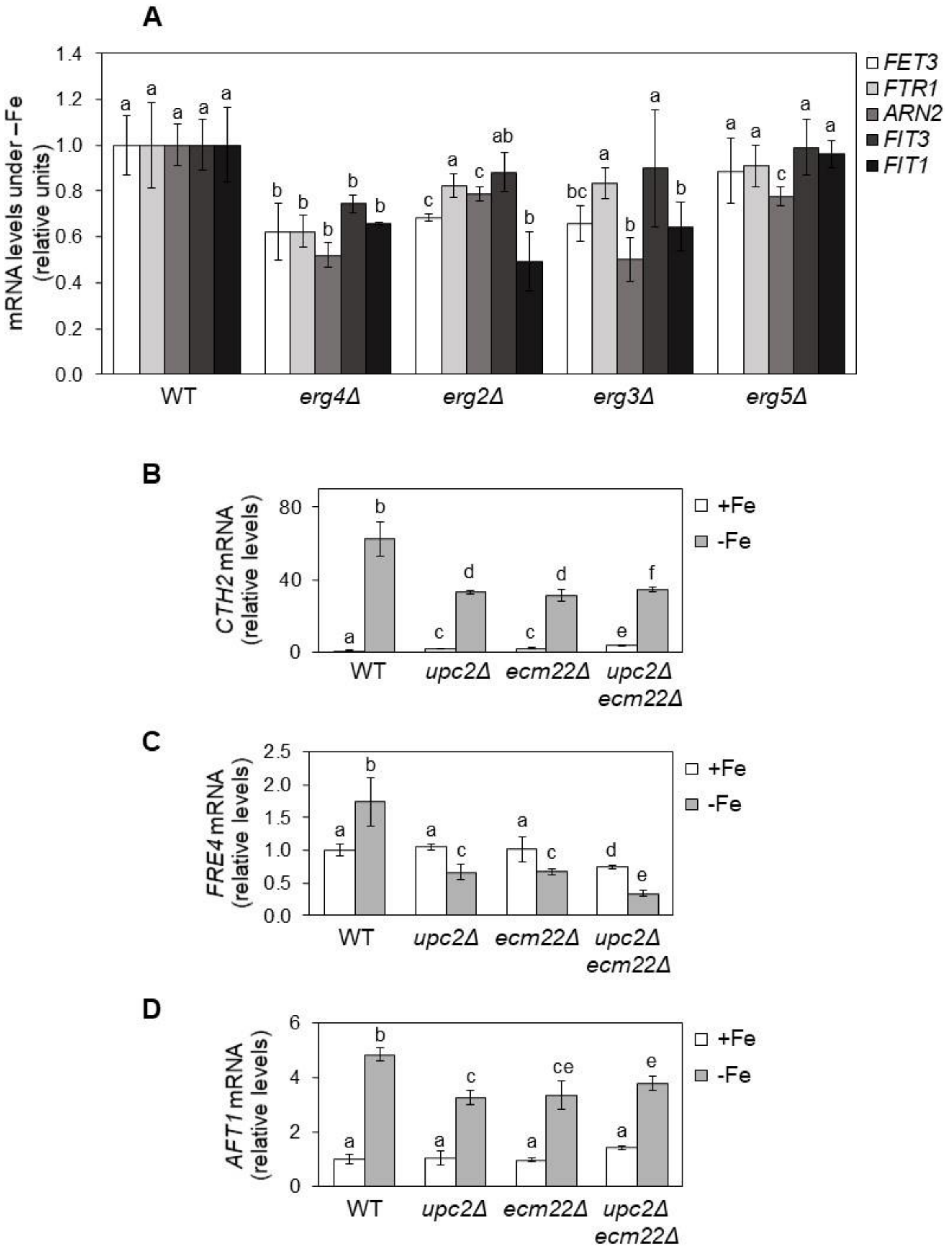

Figure S1. Mutants in genes related to ergosterol biosynthesis display defects in iron regulon activation upon iron deficiency. (A) Wild-type (WT, BY4741), *erg4Δ*, *erg2Δ*, *erg3Δ* and *erg5Δ* were cultivated as described in Figure 2A. The mRNA levels of *FET3*, *FTR1*, *ARN2*, *FIT3* and *FIT1*, normalized to *PGK1* mRNA, were determined by RT-qPCR and represented in relation to wild-type strain in -Fe. (B-D) The mRNA levels of *CTH2*, *FRE4* and *AFT1*, normalized to *ACT1* mRNA, were determined in wild-type (WT, W303), *upc2Δ*, *ecm22Δ* and *upc2Δecm22Δ* cells of Figure 2B-D and represented in relation to wild-type strain in +Fe. In all cases, data show average and SD of 3 biologically independent experiments. Different letters indicate statistically significant differences between each strain for a specific mRNA (p value < 0.05).

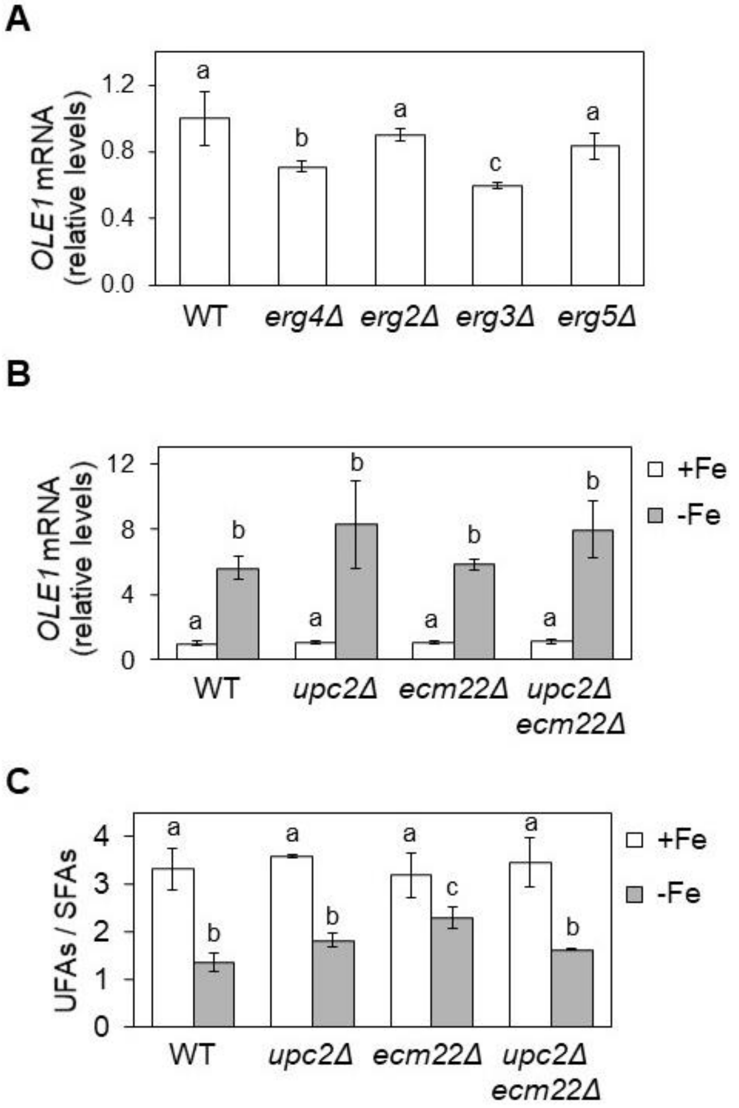

Figure S2. The limitation of iron regulon activation in cells with defects in ergosterol biosynthesis is not due to a decrease in UFA synthesis. (A) Levels of *OLE1* mRNA, normalized with *PGK1*, in wild-type (WT, BY4741), *erg4Δ*, *erg2Δ*, *erg3Δ* and *erg5Δ* cells of Figures 1 and 3A. (B) Levels of *OLE1* mRNA, normalized with *ACT1*, in wild-type (WT, W303), *upc2Δ*, *ecm22Δ* and *upc2Δecm22Δ* cells of Figure 2B-D. In both cases, data represent the average and SD of 3 biologically independent experiments, relative to WT cells in -Fe (A) or +Fe conditions (B). (C) Wild-type (WT, W303), *upc2Δ*, *ecm22Δ* and *upc2Δecm22Δ* cells were cultivated as described in Figure 4. UFA and SFA percentages were determined as described in Material and Methods, and the average and standard deviation in UFA/SFA ratio of four biologically independent experiments was represented. Different letters represent significant differences (p value < 0.05).

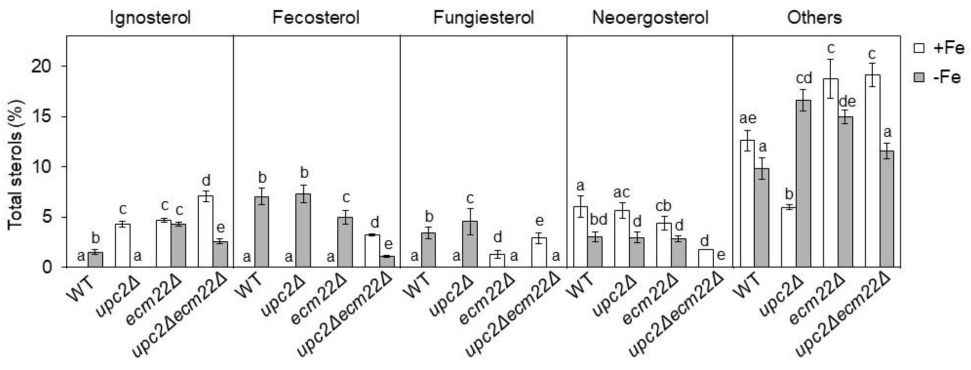

Figure S3. Iron deficiency modifies the sterols profile of Upc2/Ecm22 mutants. Relative levels of sterol intermediates, including ignosterol, fecosterol, fungisterol, neoergosterol and others, were determined in cells from Figure 4. Data were analyzed and represented as in Figure 5.

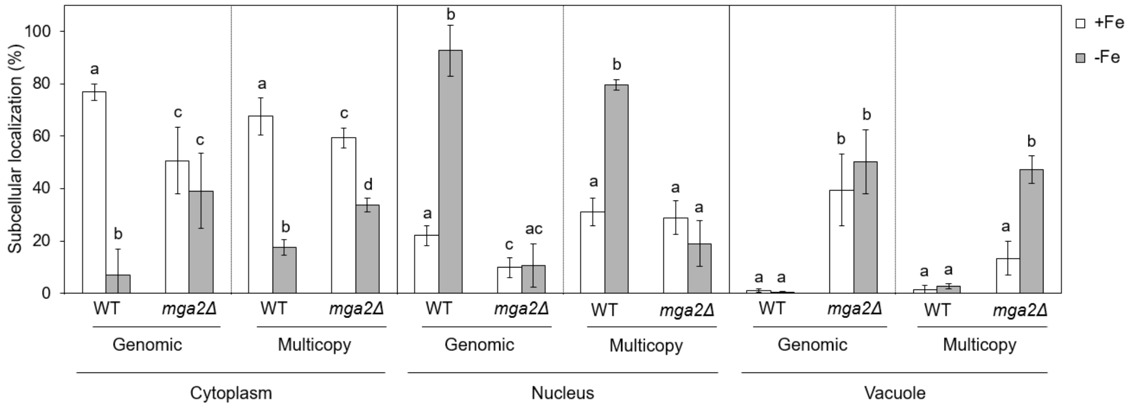

Figure S4. The localization of Aft1 protein is altered in *mga2Δ* mutants grown in iron-sufficient conditions. Wild-type (WT, BY4741) and *mga2Δ* (SPY824) cells expressing a genomic Aft1-GFP or a plasmid-expressed GFP-Aft1 (JK1346, pRS426-GFP-AFT1) were cultivated at 30 ºC for 6 hours to exponential phase in SC or in SC-Ura, respectively, without (+Fe) or with 100 μM BPS (-Fe). Quantitative analysis of Aft1 protein localization patterns were performed as described in Figure 7. Different letters represent significant differences within the same box (p value < 0.05).

Table S1. Description and source of yeast strains and plasmids used in this work.

Strain/Plasmid	Description	Source
Strains		
BY4741	*MATa his3Δ1 leu2Δ0 met15Δ0 ura3Δ0*	Invitrogen
SPY1161	BY4741 *erg2::KanMX4*	Invitrogen
SPY1162	BY4741 *erg3::KanMX4*	Invitrogen
SPY1163	BY4741 *erg4::KanMX4*	Invitrogen
SPY1164	BY4741 *erg5::KanMX4*	Invitrogen
W303-1a	*MATa ade2-1 leu2-3,112 his3-1 ura3-52 trp1-100 can1-100*	[1]
JRY7179	W303-1a *upc2::HIS3*	[1]
JRY7180	W303-1a *ecm22::TRP1*	[1]
JRY7181	W303-1a *ecm22::TRP1 upc2::HIS3*	[1]
SPY824	BY4741 *mga2::KanMX4*	Invitrogen
SPY1212	BY4741 *AFT1-GFP::His3MX6*	This study
SPY1213	BY4741 *mga2::KanMX4 AFT1-GFP::His3MX6*	This study
Plasmids		
P_{FET3}-lacZ	CEN *URA3* P_{FET3}-*lacZ*	J. Kaplan
pSP441	CEN *URA3* $P_{CYC1\text{-}FeRE}$-*lacZ*	[2]
JK1346	2 µm *URA3 GFP-AFT1*	J. Kaplan
pRS416	CEN *URA3*	M. Funk
pRS416-AFT1-1^{up}-12HA	CEN *URA3 AFT1-*1^{up}*(C291F)-12HA*	[3]
pNEV-N	2 µm *URA3*	[4]
pNEV-DAN1	pNEV-N P_{PMA1}-*AUS1*	[4]

YEp13	2 µm *LEU2*	[4]
YEp13-AUS1	YEp13 P_{PMA1}-*AUS1*	[4]
pFA6a-GFP(S65T)-His3MX6	*GFP(S65T)*-T_{ADH1}-*His3MX6*	[5]

[1] Vik, A., & Rine, J. (2001). Upc2p and Ecm22p, dual regulators of sterol biosynthesis in Saccharomyces cerevisiae. *Mol Cell Biol, 21*(19), 6395-6405. doi:10.1128/MCB.21.19.6395-6405.2001

[2] Puig, S., Askeland, E., & Thiele, D. J. (2005). Coordinated remodeling of cellular metabolism during iron deficiency through targeted mRNA degradation. *Cell, 120*(1), 99-110. doi:10.1016/j.cell.2004.11.032

[3] Ueta, R., Fujiwara, N., Iwai, K., & Yamaguchi-Iwai, Y. (2012). Iron-induced dissociation of the Aft1p transcriptional regulator from target gene promoters is an initial event in iron-dependent gene suppression. *Mol Cell Biol, 32*(24), 4998-5008. doi:10.1128/MCB.00726-12

[4] Alimardani, P., Regnacq, M., Moreau-Vauzelle, C., Ferreira, T., Rossignol, T., Blondin, B., & Berges, T. (2004). SUT1-promoted sterol uptake involves the ABC transporter Aus1 and the mannoprotein Dan1 whose synergistic action is sufficient for this process. *Biochem J, 381*(Pt 1), 195-202. doi:10.1042/BJ20040297

[5] Longtine, M. S., McKenzie, A., 3rd, Demarini, D. J., Shah, N. G., Wach, A., Brachat, A., Philippsen, P., & Pringle, J. R. (1998). Additional modules for versatile and economical PCR-based gene deletion and modification in Saccharomyces cerevisiae. *Yeast, 14*(10), 953-961. doi:10.1002/(SICI)1097-0061(199807)14:10<953::AID-YEA293>3.0.CO;2-U

Table S2. Oligonucleotides used for RT-qPCR in this work.

Name	***Sequence (from 5’ to 3’)***
FET3-qPCR-F	TGACCGTTTTGTCTTCAGGT
FET3-qPCR-R	CTCCACGATTTCATCCTTCTC
FTR1-qPCR-F	GGTCACTTGCCTTTCACCAA
FTR1-qPCR-R	TTGCTCTTCCGTCAACTCCT
ARN2-qPCR-F	TTCCTTTCGCTCCATTCAAG
ARN2-qPCR-R	GAGATACCCAGCAGCCATTT
FIT3-qPCR-F	CATCCTCTAGCACCGCTGAA
FIT3-qPCR-R	CAATAACATGACGGCAGCAA
FIT1-qPCR-F	TCTAGGGATGCCCAATCTGT
FIT1-qPCR-R	ACCAGCGGTAGTGGTTTGA
OLE1-qPCR-F	TCGACAAGAAGGGAAACGAA
OLE1-qPCR-R	CATGGTTGTTCGGAGATGTG
ACT1-qPCR-F	TCGTTCCAATTTACGCTGGTT
ACT1-qPCR-R	CGGCCAAATCGATTCTCAA
PGK1-qPCR-F	AAGCGTGTCTTCATCAGAGTTG
PGK1-qPCR-R	CGTATCTTGGGTGGTGTTCC
AFT1-F2	AATGGTGAACGGCGAGTTGAAGTAT GTGAAGCCAGAAGATCGGATCCCC GGGTTAATTAA
AFT1-R1	ATGAAAATGGACGAGAGATACGTC TAAGTTTGATTTCATCGAATTCGAG CTCGTTTAAAC
TermTEF:135F	CGACATCATCTGCCCAGAT
Aft1+275-R	CAGCCTAATCTACCGGCAAAA
